# Physical Factors Affecting Plasmid DNA Compaction in Stearylamine-Containing Nanoemulsions Intended for Gene Delivery

**DOI:** 10.3390/ph5060643

**Published:** 2012-06-18

**Authors:** André Leandro Silva, Francisco Alexandrino Júnior, Lourena Mafra Verissimo, Lucymara Fassarella Agnez-Lima, Lucila Carmem Monte Egito, Anselmo Gomes de Oliveira, Eryvaldo Socrates Tabosa do Egito

**Affiliations:** 1 Laboratório de Sistemas Dispersos (LASID), Universidade Federal do Rio Grande do Norte (UFRN), Natal/RN, Brazil; 2 Laboratório de Biologia Molecular e Genômica (LBMG), Departamento de Biologia Celular e Genética, Centro de Biociências, Universidade Federal do Rio Grande do Norte (UFRN), Natal/RN, Brazil; 3 Programa de Pós-Graduacao em Ciência e Engenharia de Petróleo, Laboratório de Analises Extratograficas, Centro de Ciências Exatas e Tecnológicas, Universidade Federal do Rio Grande do Norte (UFRN), Natal/RN, Brazil; 4 Departamento de Fármacos e Medicamentos, Faculdade de Ciências Farmacêuticas, Universidade Estadual Paulista-Unesp, Rodovia Araraquara-Jaú Km 01, 14801-902, Araraquara, SP, Brazil

**Keywords:** gene therapy, stearylamine, cationic lipid nanoemulsions

## Abstract

Cationic lipids have been used in the development of non-viral gene delivery systems as lipoplexes. Stearylamine, a cationic lipid that presents a primary amine group when in solution, is able to compact genetic material by electrostatic interactions. In dispersed systems such as nanoemulsions this lipid anchors on the oil/water interface confering a positive charge to them. The aim of this work was to evaluate factors that influence DNA compaction in cationic nanoemulsions containing stearylamine. The influence of the stearylamine incorporation phase (water or oil), time of complexation, and different incubation temperatures were studied. The complexation rate was assessed by electrophoresis migration on agarose gel 0.7%, and nanoemulsion and lipoplex characterization was done by Dynamic Light Scattering (DLS). The results demonstrate that the best DNA compaction process occurs after 120 min of complexation, at low temperature (4 ± 1 °C), and after incorporation of the cationic lipid into the aqueous phase. Although the zeta potential of lipoplexes was lower than the results found for basic nanoemulsions, the granulometry did not change. Moreover, it was demonstrated that lipoplexes are suitable vehicles for gene delivery.

## 1. Introduction

Medicine is entering a new era of treatments in which doctors will be able to treat not only symptoms, but also the cause of genetic diseases [1]. Genes influence various human diseases in general by coding abnormal proteins that are responsible for the disease or determining susceptibility to environmental agents that induce them. The development of safe and effective carriers for gene delivery has attracted enormous attention in the last decades [2,3,4,5].

Recent advances in human genomics and gene delivery systems have made it possible to cure genetic or acquired diseases using gene therapy through the direct modulation of gene expression [4,6,7,8,9,10]. In this way, gene delivery works where defective genes need to be replaced or pathogenic gene expression needs to be inhibited [9,11]. Moreover, entirely new functions can be added to cells by gene transfer [12], thereby repairing the origin of the disease [6].

Recently, the importance of nanotechnology applied to gene delivery systems [8], in which gene transfer can be achieved by viral or non-viral vehicles has been strongly emphasized [3,9,13,14,15]. Cationic nanoemulsions have been investigated as non-viral gene carriers in therapeutic gene delivery [8,10,11,16,17,18] due to the disadvantages related to the use of viral vectors (scale-up control, immunogenicity, oncogenicity, and the limited size of nucleic acid that can be packed) [8,10,19,20,21]. These systems consist of two immiscible liquids containing an oil core (natural or semi-synthetic) stabilized by co-surfactants and cationic surfactants, which are responsible for the positive charge in the droplet surface. The presence of cationic surfactants allows the complexation with the negatively charged DNA via electrostatic interactions [2,8,20,21,22,23,24], resulting in DNA compaction and consequently nanocomplex formation of emulsion/DNA [10]. Besides, cooperative hydrophobic interactions between aliphatic tails might be important in this process [24,25]. Stearylamine, a cationic lipid, is reported to be a suitable cationic surfactant for gene delivery [10,16,17].

The goal of this work was to evaluate the influence of physical factors in the formation of lipoplexes between a cationic nanoemulsion and a plasmidial DNA (pIRES2-EGFP) as a model. Several pieces of evidence lead to the supposition that different temperatures [25,32,33,34], time of complexation [11,15,16,17,20], and place of the incorporation phase of the cationic lipid into the systems [10,25,27] might have some influence on the lipoplex formation.

## 2. Experimental Section

### 2.1. Chemicals

Stearylamine, sorbitan monooleate (Span^®^ 80), and poly(oxyethylene sorbitan monooleate) (Tween^®^ 80) were obtained from Sigma Chemical (St. Louis, MO, USA). Medium-chain triglycerides (Captex^®^ 355) were donated by Abitech (Columbus, OH, USA). The water used in this study was freshly purified by a Milli-Q Gradient A10 system (Millipore, Molsheim, France). All reagents were used without further purification.

### 2.2. Plasmid PIRES2-EGFP DNA (pDNA)

The plasmid pIRES2-EGFP was amplified in *Escherichia coli* (strain DH10B) and extracted by MidiPrep [26]. The purity of the plasmidial DNA (pDNA) was measured by OD260/OD280 (1.85∼1.90; OD, optical density) and electrophoresis in a 0.7% agarose gel. pDNA was resuspended in pure water (Milli-Q) and frozen in aliquots.

### 2.3. Preparation of Cationic Lipid Nanoemulsions (CLNs)

The nanoemulsions were produced by the sonication method. Briefly, the aqueous phase components were weighed and mixed with pure water (Milli-Q). The mixture was sonicated for 3 minutes using a probe sonicator at 30 W (VCX600, Sonics and Materials, Danbury, CT, USA). This mixture was added into the oily phase, which was 50 ng of Captex^®^ 355 per mg of nanoemulsion, and sonicated for 4 min in an ice-water bath and, then, stored at 4 ºC. In order to evaluate possible changes in the physicochemical properties of the nanoemulsions containing stearylamine (CLN) and consequently variations in the complexation efficiency, two different CLNs were formulated, in which the cationic lipid stearylamine was dispersed into the water phase (CLN_1_) or into the oily phase (CLN_2_), as described in Table 1.

**Table 1:** Composition of the CLNs: CLN with stearylamine dispersed into aqueous phase (CLN_1_) or CLN with stearylamine dispersed into oily phase (CLN_2_).

Component	CLN_1_ %_(w/w)_	CLN_2_ %_(w/w)_
**Oily phase**		
Captex^®^ 355 (TCM)	5	5
Span^®^ 80	0.8	0.8
Stearylamine	-	0.16
**Aqueous phase**		
Tween^®^ 80	1.20	1.20
Stearylamine	0.16	-
Water qsp.	100	100

### 2.4. Particle Size and Zeta Potential Analysis

The mean particle size and zeta potential of the CLN and lipoplexes (at the best pDNA/CLN ratio, which was 2.3 (2.3 ng of pDNA per 1 ng of CLN_1_) and 0.92 (0.92 ng of pDNA per 1 ng of CLN_2_, found by the compaction process assay)) were measured by Dynamic Light Scattering (DLS) and phase analysis light scattering, respectively (Brookhaven ZetaPALS, Holtsville, NY,USA). The formulations were diluted with distilled water by 300-fold before the measurement. 

### 2.5. Preparation of PDNA/CLN Complexes

#### 2.5.1. Influence of the Stearylamine Loading Process at the Water or Oil Phase on the PDNA Compaction Process

To analyze the efficiency in the compaction process of the formulations, both CLN_1_ and CLN_2_ were left in contact with pDNA (460 ng/well or 4.60 ng of pDNA per ng of oil phase of CLN_1 _in the case of pDNA/CLN ratio 4.60) for 30 min at room temperature (RT – 25 ± 2°C) at increasing amounts of CLN ranging from 5 µL to 50 µL. After the preliminary results from such CLN volume variation, a screening was done at a narrow interval from 2 µL to 10 µL (Table 2). Although the volume inside the well varied, the pDNA concentration remained constant at 460 ng/well. In fact, because the full lipoplex volume will be used for the gel running and the volume variation did not affect the pDNA state by itself, the gel running would present the same bend intensity if no lipoplex were formed. It is important to note that CLN has 5% (w/w) of oil. This corresponds at 50 ng of Captex^®^ 355 per 1 mg (or 1 µL) of nanoemulsion. Because the oil content has a direct relationship with the number of droplets into the CLN system, and by consequence the state in which the pDNA was found into the media, this value was used to establish the pDNA/CLN ratio. 

**Table 2:** Study concentration of CLN and its corresponding pDNA/CLN ratio.

CLN (μL)	pDNA(ng)/CLN(ng oil phase) ratio
**2**	4.60
**4**	2.30
**5**	1.84
**6**	1.53
**7**	1.31
**8**	1.15
**9**	1.02
**10**	0.92

#### 2.5.2. Influence of the Time of Complexation

Because CLN_1_ presented a better compaction process compared to CLN_2_, different times of contact between pDNA and CLN_1_ (at the best pDNA/CLN_1_ ratio, found by the compaction process assay) were assessed at 0, 15, 30, 60, 90, and 120 min. This experiment was conducted at RT.

#### 2.5.3. Influence of the Temperature on the Lipoplex Formation

To analyze the influence of temperature in the compaction process assay, three different temperatures were evaluated: RT, 37 ± 2 °C and 4 ± 1 °C (ice-bath). Again, CLN_1 _formulation was used as the reference due to the results obtained in the compaction process assay. pDNA (4.60 ng of per ng of internal phase of CNL_1_) was mixed and left in contact for 30 min.

### 2.6. Efficiency of Complexation by Electrophoresis Assay

The complexes were loaded onto 0.7% agarose gel containing ethidium bromide (0.5 mg/mL) using 40 mM Tris acetate and 1mM EDTA as buffer. Gel was run at 80 mV for 60 min and the trapping efficiency of pDNA by CLNs was determined as the DNA was no longer accessible to ethidium bromide intercalation by the assay of gel electrophoresis [20].

## 3. Results and Discussion

### 3.1. Particle Size and Zeta-Potential Characteristics

DLS analysis of CLNs and lipoplexes ensured that the produced systems were at nanoscale dimensions (Table 3). It is important to note that the pDNA in stock solution presented a mean particle size of 52.2 ± 0.6nm. It should be emphasized that this size is given as a purpose of comparison with the CLN size. In fact, hydrodynamic volume (which is measured by the DLS) changes according the kind of coiled in which the plasmid has been presented. 

**Table 3:** Characterization (Size, Polydispersity Index (PI) and Zeta Potential) of CLNs and lipoplexes (CLN_1 _– 1.60 pDNA/CLN_1 _ratio; CLN_2_ – 0.64 pDNA/CLN_2 _ratio).

Formulation	Size (nm) ± SD	PI (± SD)	Zeta Potential (mV) ± SD
**CLN_1_**	195.1 ± 2.9	0.185 ± 0.015	41.9 ± 4.3
**CLN_1_-lipoplex**	200.7 ± 4.2	0.177 ± 0.027	22.5 ± 3.4
**CLN_2_**	199.1 ± 4.2	0.185 ± 0.004	39.6 ± 4.1
**CLN_2_-lipoplex**	204.0 ± 0.2	0.213 ± 0.010	25.7 ± 0.3

CLNs presented particle sizes smaller than 200 nm, for both CLN_1_ and CLN_2_. After lipoplex formation, no significant increase in droplet size was found for either CLN_1_ or CLN_2_, probably because the pDNA was not only adsorbed, but also condensed on the droplet surface, and its negligible concentration (460 ng) does not induce enough surface volume charge to improve the nanoemulsion droplet size.

The Polydispersity Index (PI) was around 0.2 for all formulations (Table 3). Moreover, the droplet size distribution of the systems was quite homogeneous and not influenced by the lipoplex formation generated between pDNA and CLN. 

As can be observed by the positive values of zeta potential, stearylamine gives a positive charge to the droplet surface. Zeta potential results were around +40 mV for both CLN_1_ and CLN_2_, which not only provides enough positive charges for lipoplex formation, but also provides CLN stability by electrostatic repulsion. When lipoplexes were formed, zeta potential decreased (Table 3). This could be explained by the negative charge from the pDNA being added to the system, thereby turning the zeta potential less positive.

This result is quite interesting because although the complexation study revealed that CLN_1_, which has stearylamine at the outermost oil/water interface, possesses different behavior concerning lipoplex formation compared to CLN_2_, which has stearylamine at the innermost oil/water interface, both products presented the same zeta potential. This is probably due to the migration of the stearylamine from the innermost to the outermost interface induced by the dilution process of 300 fold, mandatory for this assay.

### 3.2. Effects of Stearylamine Loading Phase on the pDNA/CLN Complexation

Although at different pDNA/CLN ratios, 100% of DNA compaction was achieved by diluting the pDNA solution with both CLN_1_ and CLN_2_. The presence of the CLN droplets at pDNA/CLN ratio above 2.30 for CLN_1 _and above 0.92 for CLN_2_ (Table 2), in which a high amount of droplets exists, induces totally complex formation and no free pDNA could be seen in the gel bend. Complexation is possible due to electrostatic interactions between the positively charged stearylamine head group (which is located on the oil/water interface) and the negatively charged phosphate anchor from the pDNA [5,6,8,16]. 

**Figure 1:**
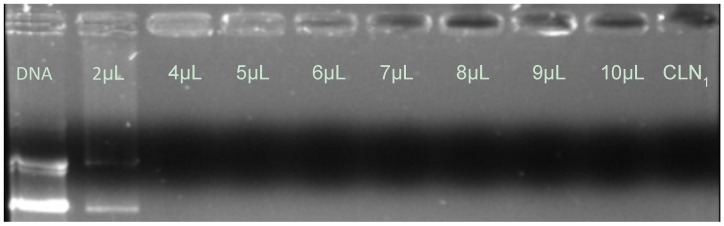
Influence of the CLN_1_ amount on the compaction of pDNA.

Matulis *et al.* have demonstrated by thermodynamic assays that condensation is dependent on DNA concentration [25]. In this way, from the data set revealed by CLN_1_, 4 μL of CLN_1_ was enough to completely condense the genetic material. Figure 1 shows that by increasing the DNA/CLN ratio higher than 2.30 pDNA/CLN_1_ ratio, no free pDNA can be seen, which means that the 460 ng of pDNA was completely condensed with 4 µL of CLN_1_.

It is known that polyethylene glycol (PEG) domains present in Tween^℘^ 80 avoid oil droplet aggregation in emulsions [10]. This is also related to a steric effect (because of those mentioned PEG domains) for Tween^℘^ 80, which could make the entrapment of the pDNA molecule [27] difficult. When stearylamine is placed into the aqueous phase, Tween 80^℘^ might be placed on the oil/water surface and the stearylamine could be located more externally at the outermost surface of the drop, minimizing the furtive effect from PEG. On the other hand, when stearylamine is loaded into the oil phase, the charge is there, but located in the innermost regions of the drop, which allows the steric effect from PEG to participate, and makes the complete complexation of pDNA difficult. It is important to note that, in this case, a probable partition of stearylamine into the two phases was negligible because the steric effect, which is dependent on the phase where stearylamine was placed, was demonstrated. This is in agreement with the works of Matulis *et al.*, who described a binding model where cationic head groups are near DNA phosphates while the aliphatic tails stand perpendicular to the DNA surface [25]. 

**Figure 2:**
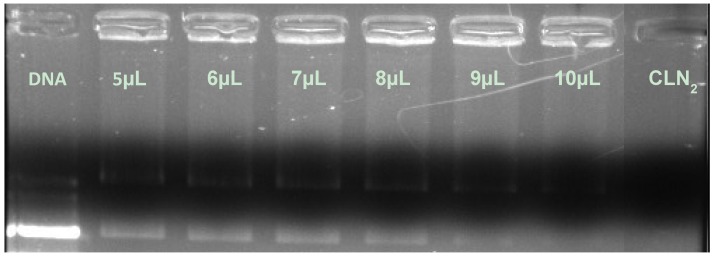
Influence of the CLN_2_ amount on the compaction of pDNA.

Concerning CLN_2_, it was found that a larger amount of CLN was necessary to compact the same quantity of pDNA compared to CLN_1_ (Figure 2). In this case, around 10 μL of CLN_2_ (0.92 pDNA/CLN_2_ ratio) was necessary to completely compact the 460 ng of the genetic material, which represents more than twice the volume of CLN_1_. 

Most of the time, complexation is done by adsorption of the genetic material to the cationic system by electrostatic interaction [16,17,19,20,21]. However, other methods could be used to ensure that the genetic material is loaded on the carrier. Nanoencapsulation of genetic material is another related approach [28]. This was demonstrated by Badea *et al.* [29], who produced nanoparticles where the genetic material was put into the formulation during the manufacturing process. Also, Kwon *et al.* [23] previously prepared nanoemulsions, added pDNA, and sonicated the mixtures in order to form lipoplexes. All these approaches are in agreement with our results. Moreover, a combination of two mechanisms, named electrostatic interaction and nanoencapsulation assembly, can play an important role in the pDNA complexation in CLN systems.

### 3.3. Time Effect on pDNA/CLN Complexation

Concerning non-viral gene delivery systems, the literature reports different times of complexation. It seems that there are no rules governing this variation and many different times are related to the complete complexation of genetic material. Time of complexation changes not only concerning the kind of genetic material, but also the different pharmaceutical systems. Tang *et al.* [20] worked with two complexation times. They started complexing for 30 min and then reduced the time for 10 min of incubation at RT. Kim *et al*. [14] did nanoparticle complexing of pDNA for 1 hour at RT but Munier *et al*. [19] used 10 min as the time of complexation for their nanoparticles. Other authors working with nanoparticles have described achieving complete complexation in just 5 min [2,3,21]. Teixeira *et al*. [16] associated oligonucleotides with cationic nanoemulsions for 12 h. Martini *et al*. [17] used a similar system and associated the genetic material for 30 min and Bruxel *et al.* [11] for just 15 min. Concerning liposomes, there is also no pattern of complexation time [5,30,31]. Zhang *et al.* [15] evaluated complexation of both pDNA and siRNA for 20 min using a unique system and found different ratios of complexation according to the kind of genetic material. Therefore, it could be seen that each researcher chooses the best time of association experimentally, according to the kind of both formulation and genetic material [11,15,16,17,20].

**Figure 3:**
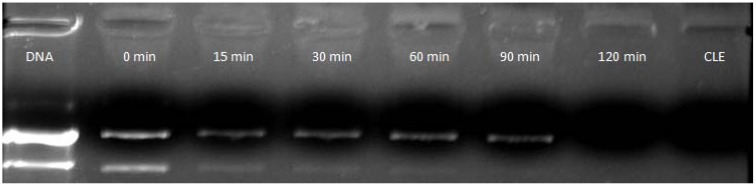
Time complexation of pDNA by CLN_1_ (2.30 pDNA/CLN_1_ ratio).

This study reveals that for CLN 120 min is the best time of complexation (Figure 3). It was observed that after just adding the pDNA in contact with the CLN, lipoplexes were immediately generated in spite of the important amount of DNA that remained uncomplexed. With the time increasing, the intensity of the bend diminished and, after 120 min, free pDNA was no longer observed, meaning that 100% of the pDNA was complexed and condensed.

### 3.4. Effects of the Environmental Temperature on the pDNA/CLN Complexation

**Figure 4:**
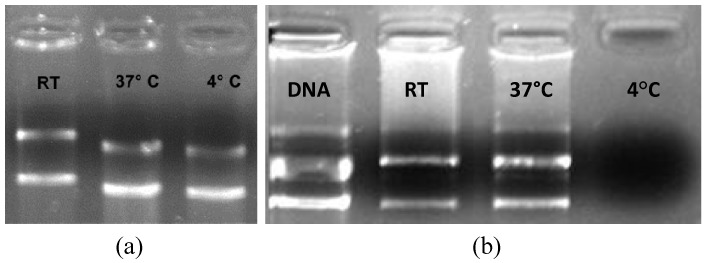
Influence of the environmental temperature on (a) naked pDNA (1 µg per well, with a total mass of pDNA of 1,000 ng) and (b) pDNA/CLN_1_ complex formation (158.75 ng of pDNa per µL of CNL_1 _, with a total mass of pDNA of 1,270 ng).

Usually, complexation is done at RT [14,16,17,19,20]. In this study a comparison of three different temperatures [RT, “cold” temperature (4 °C), and “hot” temperature (37 °C)] was performed to observe whether there is any influence of temperature on the complexation efficiency (Figure 4). It was observed that the same amount of pDNA in the same time of complexation had differences in the complexation range. Naked pDNA was also analyzed at the three different temperatures, as a control, to ensure that there would be no difference in the gel visualization when the pDNA was maintained at different temperatures.

As shown in Figure 4, at RT some compaction is achieved. However, it seems that by increasing the temperature to 37 °C less pDNA is packed. On the other hand, at 4 °C all the genetic material was packed by the CLN_1_. A crucial point is that at this temperature, naked DNA, although at lower mass concentration (1,000 ng), presented a bend on the running gel (Figure 4a). The reason is that, according to the temperature, plasmids should assume, more or less, the supercoiled form [32,33]. In fact, at high temperature, the pDNA assumes more relaxed forms and gains more volume. Inversely, at cold temperature it assumes less volume and becomes supercoiled [34] and more pDNA can be packed by the CLN. Furthermore, Matulis *et al.* demonstrated that at a higher temperature, the interaction between the cationic lipids with DNA is slightly weaker, due to the less favorable electrostatic and hydrophobic entropies and electrostatic enthalpy [25]. For a clinical approach, it is important to note that the transfection efficiency is highly dependent on the quality and conformation of the used DNA [35].

## 4. Conclusions

The use of non-viral gene delivery systems for gene therapy can solve most of the drawbacks related to viral vectors such as any serious side effects related to its safety and the host immune and inflammatory responses. However, clinical application of this approach requires the development of safe and efficient delivery vehicles. 

Developing non-viral gene delivery systems is not an easy task and a large number of variations can influence the final pharmaceutical and nanotechnological systems. Despite the fact that models of the interactions between cationic lipids and genetic materials have been developed, this process is still poorly understood. In this work it has been shown that temperature, cationic lipid phase incorporation, and time of complexation all play important roles in the formation of lipoplexes. In fact, these parameters could block the genetic carrier from complexing with the pDNA in order to form lipoplexes, which is mandatory for gene delivery. Moreover, the results presented here allow us to speculate that the stearylamine containing cationic nanoemulsions would be a suitable non-viral vehicle for gene therapy. However, further studies such as cytotoxicity, *in vitro*, and *in vivo* transfection assays are necessary to confirm this hypothesis.
